# Sensitivity Analysis of Cracking Behavior in Fully Ceramic Microencapsulated Fuel

**DOI:** 10.3390/ma19142938

**Published:** 2026-07-08

**Authors:** Shichao Liu, Haoyue Huang, Chi Chen, Yanli Zhao, Yuanming Li, Chenxi Li, Yi Zhou

**Affiliations:** National Key Laboratory of Nuclear Reactor Technology, Nuclear Power Institute of China, Chengdu 610200, China

**Keywords:** fully ceramic microencapsulated fuel (FCM fuel), crack, sensitivity analysis, residual pore

## Abstract

To identify the key factors influencing the cracking behavior of fully ceramic microencapsulated (FCM) fuel, this study employed the MOOSE V1.3 multiphysics coupling platform and the cohesive phase-field fracture theory to simulate crack initiation and propagation in FCM fuel, with particular attention to the effects of particle spacing and residual pore in the matrix. Results showed that during early irradiation stages, in the absence of matrix defects, particle spacing had minimal influence on the distribution of the maximum principal stress. However, when residual pore was present in the SiC matrix, significant stress concentration occurred at the porosity sites, where the maximum principal stress was localized. Smaller particle spacing promoted crack initiation in the SiC matrix between adjacent particles and led to a higher number of cracks under the same fast neutron fluence. In the presence of residual pore, crack nucleation occurred at porosity sites even at low neutron fluence; at a fluence of 2.3 × 10^25^ n/m^2^, through-thickness cracks formed in FCM fuel containing residual pore, resulting in the loss of fission product containment capability.

## 1. Introduction

Fully Ceramic Microencapsulated (FCM) fuel consisted of TRISO fuel particles randomly dispersed within a SiC matrix. The TRISO fuel particle was composed of fuel kernel, Buffer layer, inner dense pyrolytic carbon (IPyC) layer, silicon carbide (SiC) layer, and outer dense pyrolytic carbon (OPyC) layer, arranged sequentially from the inside out. The SiC layer served as the primary structural layer, effectively restricting the release of radioactive fission products, withstanding the internal pressure generated by fission gases, and ensuring the safety and integrity of the TRISO particle [1,2]. The Buffer layer, characterized by its lower density, acted as the primary accommodation layer for fission products produced in the kernel, and its thickness determined the fuel burnup. During irradiation, the IPyC and OPyC layers initially underwent volumetric shrinkage, exerting compressive stress on the SiC layer to protect its integrity. Additionally, the OPyC layer prevented abrasion and damage to the SiC layer during manufacturing. Compared to UO_2_ fuel, FCM fuel exhibited superior fission product retention capability under beyond-design-basis conditions, excellent resistance to high-temperature steam corrosion, and a longer accident response time, making it a significant candidate for accident-tolerant fuels in light water reactors [3].

The simulation of in-reactor behavior was a primary means of predicting the performance of FCM fuel. However, existing simulations employed boundary conditions and representative volume element settings that introduced significant simplifications, failing to fully reflect the actual in-reactor behavior of FCM fuel. Petti et al. [4] posited that under steady-state operating conditions, the stress in the SiC layer originated from three main sources: outward tensile stress caused by internal pressure, and inward compressive stresses induced by the IPyC and OPyC layers. Their results indicated that fuel kernel dimensions, coating layer thickness, and temperature all significantly influenced the stress distribution in the SiC layer. Building upon the TRISO particle model, Ougouag et al. [5] incorporated the effect of the SiC matrix on individual TRISO particles by adding a layer of SiC matrix of specific thickness to the exterior of the TRISO particle. Their results demonstrated that the SiC matrix significantly affected the stress state of the TRISO coating layers. Variations in the structural dimensions of the TRISO particle led to changes in the stress state of the FCM fuel; neglecting the outermost OPyC layer or increasing the thickness of the SiC matrix resulted in a sharp increase in stress within the SiC layer. To reduce computational cost, Schappel [6] calculated the SiC matrix and TRISO particles separately, ignoring the interaction between the matrix and the particles. It was observed that the stress state of the SiC matrix changed significantly between the beginning of life and the end of life.

Superior fission product retention capability was a critical indicator for evaluating FCM fuel. The initiation and propagation of cracks within FCM fuel had a significant impact on its integrity and failure modes. Investigating the mechanisms of crack initiation and propagation was of great scientific and engineering significance for understanding fuel failure modes and optimizing the FCM fuel structure. However, there were limited domestic and international studies on crack initiation in FCM fuel. Terrani et al. [7,8] experimentally investigated the initiation of matrix cracks and their interaction with TRISO particles. They found that when matrix cracks in FCM fuel propagated to the OPyC layer, deflection occurred, causing the cracks to bypass the TRISO particles and continue extending through the matrix. Although cracks originating at TRISO particles could lead to SiC layer failure, no significant crack propagation was observed in the SiC matrix between adjacent TRISO particles. No studies have been conducted on the computational methods for the cracking behavior of FCM fuel. Kamalpour et al. [9] employed the extended finite element method to analyze crack propagation in the SiC matrix of FCM fuel under typical operating conditions and conducted a sensitivity analysis by modifying the swelling of pyrolytic carbon; however, their study focused on the cracking of the SiC layer within TRISO particles. Collin et al. [10] investigated the failure behavior of UN-kernel TRISO particles, showing that in a pressurized water reactor environment, TRISO particles could meet a 1400-day operational lifespan, with the primary failure mode being the rapid increase in SiC layer stress caused by re-contact between the IPyC and Buffer layers at high burnup; however, that study did not address cracking or crack propagation in the coatings or the matrix. Chen et al. [11] used COMSOL-5.1 software to perform a three-dimensional multi-physics coupling analysis of FCM fuel, simulating the thermo-mechanical behavior of UO_2_-TRISO particles under steady-state operation and evaluating failure probability, yet they did not investigate the cracking behavior of FCM fuel. While existing studies have extensively investigated the failure probability and stress state of FCM fuel using finite element methods, research on its cracking failure behavior remains limited. Consequently, current approaches cannot accurately evaluate the cracking modes and thresholds of FCM fuel. This study investigates the cracking behavior of FCM fuel by analyzing the stress states of the SiC coating layers and the SiC matrix, and by proposing cracking criteria for both components. This work addresses the gap in the existing literature regarding the analysis of FCM fuel cracking behavior.

In summary, despite the unique multi-layer complex structure and interfaces of FCM fuel, there were few studies on its cracking behavior, and a lack of sensitivity analysis regarding matrix defects. Based on the MOOSE V1.3 phase-field fracture simulation program, this paper analyzed the cracking and crack propagation behavior of FCM fuel elements under typical operating conditions. Sensitivity analyses were performed regarding different TRISO particle spacings and matrix pore defects. The study clarified the cracking mechanisms and modes of FCM fuel, laying a foundation for the safety performance evaluation of FCM fuel.

## 2. Calculation Method

For heterogeneous materials, the interfacial strength often differed from that of the bulk material, thereby influencing crack propagation paths and causing phenomena such as crack deflection. FCM fuel contained multiple material interfaces, including the fuel kernel/Buffer, Buffer/IPyC, IPyC/SiC, and SiC/OPyC interfaces. To describe the interface location, an interface variable (β), analogous to a phase-field variable, was introduced in this study: β:β = 1 indicated the interface center, β = 0 represented the bulk material region, and 0 < β < 1 denoted the transition region. The governing equation for the interface variable was given by [12,13]:

(1) $$l_{i}^{2}\Delta \beta =\beta$$

The boundary conditions were defined as *β* = 1 at the interface center and zero flux at the computational domain boundaries: ∇β⋅n=0. The parameter $l_{i}$ controlled the width of the interface. For a one-dimensional geometry, the analytical solution of the equation was [14]:

(2) $$\beta =e^{-\frac{x}{l_{i}}}$$

After “diffusing” the interface description, the physical properties of the entire computational domain were described uniformly using a weighted average method to obtain the fracture strength and critical energy release rate [14]:

(3) $$\bar{\sigma_{f}}=\beta (x)\sigma_{f}^{i}+\left[1-\beta (x)\right]\sigma_{f}^{b}$$

(4) $$\bar{G_{c}}=\beta (x)G_{c}^{i}+\left[1-\beta (x)\right]G_{c}^{b}$$

For the calculation of FCM fuel cracking, the governing equation based on the Cohesive Phase Field Fracture theory adopted in this paper was [15]:

(5) $$\frac{3}{4}G_{c}l_{0}\Delta d=\frac{3G_{c}}{8l_{0}}d+g^{\prime }(d)\Psi (\varepsilon )$$

where $M=\frac{3G_{c}}{8l_{0}}$, and *p* was an integer greater than or equal to 1; in this study, *p* = 2. Analysis of Equation (5) indicated that cracking occurred only when the strain energy density exceeded $\Psi (c)=-\frac{M}{\omega^{\prime }(0)}$.

This study implemented a standardized finite element discretization of Equation (5) based on the MOOSE V1.3 platform. First, the weak form was derived. The weak form of the first term on the left-hand side was [16]:

(6) $$a(d,v)=l_{0}^{2}\int_{\Omega }(\nabla v\cdot \nabla d)dV$$

The weak form of the second and third terms on the right-hand side was:

(7) $$f(d,v)=\frac{1}{2}\int_{\Omega }\left(v,d\right)dV+\frac{4l_{0}}{3G_{c}}\Psi (\varepsilon )\int_{\Omega }\left(v,g^{\prime }(d)\right)dV$$

where *v* was the test function.

Material crack propagation altered the stress state; therefore, after calculating the phase-field fracture variable, it was necessary to update the stress tensor. The strain energy density Ψ(ε) was decomposed into two parts, $\Psi^{+}(\varepsilon )$ and $\Psi^{-}(\varepsilon )$, corresponding to tensile and compressive effects, respectively. Decomposition methods for strain energy density included spherical-deviatoric decomposition and spectral decomposition. Since FCM fuel primarily underwent brittle fracture, the principal strain spectral decomposition method was used to decompose the strain tensor [17]:

(8) $$\varepsilon =\sum_{a=1}^{3}\varepsilon_{a}n_{a}⊗n_{a}$$

where {*ε_a_*}*a* = 1, 2, 3 were the principal strains, {*n_a_*}*a* = 1, 2, 3 were the principal strain directions, and *n_a_* was the eigenvector of the strain tensor. The strain energy density components $\Psi^{+}(\varepsilon )$ and $\Psi^{-}(\varepsilon )$ were calculated as follows:

(9) $$\Psi^{\pm }(\varepsilon ):=\frac{\lambda }{2}\langle \varepsilon_{1}+\varepsilon_{2}+{\varepsilon_{3}\rangle }_{\pm }^{2}+\mu \left({\langle \varepsilon_{1}\rangle }_{\pm }^{2}+{\langle \varepsilon_{2}\rangle }_{\pm }^{2}+{\langle \varepsilon_{3}\rangle }_{\pm }^{2}\right)$$

where *λ* was the first Lamé constant, *μ* was the second Lamé constant, and $\langle \varepsilon_{1}+\varepsilon_{2}+\varepsilon_{3}\rangle$ was the volumetric strain. According to the relationship between stress and strain energy density, the stress tensor was obtained as:

(10) $$\sigma_{0}^{+}:=\sum_{a=1}^{3}\left[\lambda \langle \varepsilon_{1}+\varepsilon_{2}+{\varepsilon_{3}\rangle }_{\pm }+2\mu {\langle \varepsilon_{a}\rangle }_{\pm }\right]n_{a}⊗n_{a}$$

## 3. Geometric Model and Material Properties

### 3.1. Geometric Model

**Figure 1:**
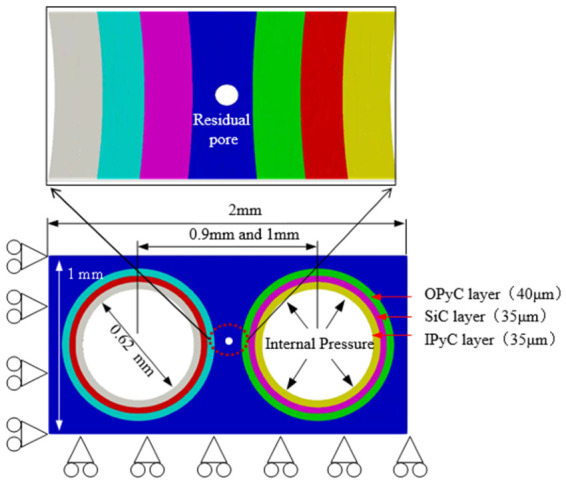
Computational model for cracking behavior of FCM fuel.

To reduce computational cost, this study employs a two-dimensional plane stress assumption and simulates the cracking and crack propagation behavior of FCM fuel under typical operational conditions using the MOOSE V1.3 phase-field fracture module. As shown in Figure 1, two TRISO particles with inter-particle spacings of 0.9 mm and 1 mm were considered. To investigate the influence of matrix defects on cracking behavior, residual pore with a diameter of 0.3 mm were introduced into the matrix. Based on literature simulation results and post-irradiation examination data of TRISO particles, the Buffer layer and the inner pyrolytic carbon (IPyC) layer separate during irradiation, and there is no interaction between the Buffer layer and the other coating layers. Consequently, the fuel kernel and the Buffer layer are excluded from the calculations in this study. This work assumes tight bonding between the various coating layers with no transitional layers present, which is consistent with reported findings [18]. Since the Buffer layer separates from the IPyC layer during irradiation and no interaction occurs between the Buffer layer and other coating layers, the fuel kernel and Buffer layer were excluded from the computation. It is assumed that all layers are perfectly bonded with no interfacial transition zones. Although an interface variable with diffusive characteristics is employed to model the interface, only the critical energy release rate and fracture strength are affected by the interface; other properties, such as Young’s modulus, remain unchanged. During the calculations, the influence of interfaces on the stress state of FCM fuel was considered. The interfacial fracture strength and critical energy release rate were introduced to describe crack propagation behavior at the FCM fuel interfaces, thereby ensuring crack deflection at the interfaces.

For the crack sensitivity analysis, three distinct cases were defined, with detailed structural configurations listed in Table 1. The analysis primarily focuses on the sizes of the two TRISO particles and the influence of matrix pore defects.

The boundary conditions were set as follows: the left and bottom edges were symmetric boundaries, and the inner side of the IPyC layer was subjected to internal pressure from fission gas. Structured meshes were used for finite element discretization. The characteristic length of the computational model was set to 0.006 mm, and the characteristic width of the interface was set to 0.003 mm. Due to the small geometric dimensions of the computational model, the material temperature distribution was relatively uniform; therefore, the temperature of all materials was set to the typical operating temperature of 1500 K, with a fast neutron flux of 5 × 10^17^ n/m^2^/s. It was assumed that the fission gas internal pressure was proportional to time, with a pressurization rate of 8 MPa/10^7^ s.

### 3.2. Materials Properties

The density of the SiC layer and matrix in FCM fuel were set as 3.18 g/cm^3^. The elastic modulus and thermal expansion coefficient were given by [19]:

(11) $$E_{SiC}=460-0.04T\exp\left(-\frac{962}{T}\right)$$

(12) $$\alpha (\times 10^{-6})=\left\{\begin{array}{lll} -1.8267+0.0178T-1.5544 & & \\ \times 10^{-5}T^{2}+4.5246\times 10^{-9}T^{3} & & T\leq 1200\;K\\ 5.0 & & T>1200\;K \end{array}\right.$$

Irradiation swelling in SiC materials was caused by the accumulation of Frenkel dislocation loops and voids. At temperatures above 800 °C, swelling was induced by voids formed through vacancy migration and coalescence, while above 1250 °C, it was caused by void agglomeration. The irradiation swelling of SiC materials at high temperatures was expressed as [19]:

(13) $$\frac{\Delta V}{V}=\left\{\begin{array}{ll} (-1.3528\times 10-5T+0.015329)\left[1-\exp\left(-\frac{\phi }{0.3396}\right)\right] & 1073\;K\leq T\leq 1273\;K\\ 0.0018\left[1-\exp\left(-\frac{\phi }{0.3396}\right)\right]+0.001297\phi & 1523\;K\leq T \end{array}\right.$$

where the temperature *T* was in Kelvin, and *ϕ* was the fast neutron fluence (10^25^ n·m^−2^).

Due to the excellent high-temperature resistance of SiC, the influence of thermal creep was not considered in the actual calculation process; typically, only the irradiation creep of SiC was taken into account [20]:

(14) $$\overset{\cdot }{\varepsilon }=K\sigma \overset{\cdot }{\phi }$$

where *K* was the irradiation creep constant (MPa/n/m^2^), *σ* was the equivalent stress (MPa), and $\overset{\cdot }{\phi }$ was the fast neutron flux rate (10^25^ n·m^−2^·s^−1^). According to the literature, an equivalence of one displacement per atom (dpa) = 1 × 10^25^ n/m^2^ (E > 0.1 MeV) is assumed in the following discussion [19].

The PyC layer had a significant influence on the stress state of the SiC layer and SiC matrix, thereby affecting their cracking status. For the PyC layer, primary attention was paid to its elastic modulus, irradiation deformation, and creep. According to the PARFUME model, the elastic modulus of PyC was anisotropic, with specific expressions as follows:

(15) $$\begin{array}{c} E_{r}=25.5(0.384+0.324\rho )(0.481+0.519BAF)\\ (1+0.23\Phi )\left[1+0.00015(T-20)\right] \end{array}$$

(16) $$\begin{array}{c} E_{t}=25.5(0.384+0.324\rho )(1.463-0.463BAF)\\ (1+0.23\Phi )\left[1+0.00015(T-20)\right] \end{array}$$

where *ρ* was the density (g/cm^3^), BAF was the anisotropy factor, *Φ* was the fast neutron fluence (10^25^ n·m^−2^, E > 0.18 MeV), *T* was the temperature (°C), and *E_r_* and *E_t_* were the radial and tangential elastic moduli (GPa), respectively.

The irradiation strain equations for the PyC layer in the radial and tangential directions were [20]:

(17) $${\dot{\varepsilon }}_{r}=-0.077\exp(-\Phi )+0.031$$

(18) $${\dot{\varepsilon }}_{\theta }=-0.036\exp(-2.1\Phi )-0.01$$

The radial irradiation creep equation for the PyC layer was presented below, and the irradiation creep strains in other directions could be deduced by analogy:

(19) $${\dot{\varepsilon }}_{cr,r}=K_{pyc}[\sigma_{r}-\nu_{c}(\sigma_{\theta }+\sigma_{\phi })]\dot{\Phi }$$

where $\dot{\Phi }$ was the fast neutron fluence rate (10^25^ n·m^−2^·s^−1^); ${\dot{\varepsilon }}_{cr,r}$ was the component of the creep strain rate (s^−1^) in the radial direction; *v_c_* was the creep Poisson’s ratio; and *K_pyc_* was the temperature-dependent creep coefficient.

The critical energy release rate and fracture strength of the materials and interfaces significantly influenced the cracking behavior of FCM fuel. The critical energy release rate and fracture strength involved in this study showed in Table 2 [21].

## 4. Results and Discussion

### 4.1. Effect of Particle Spacing

**Figure 2:**
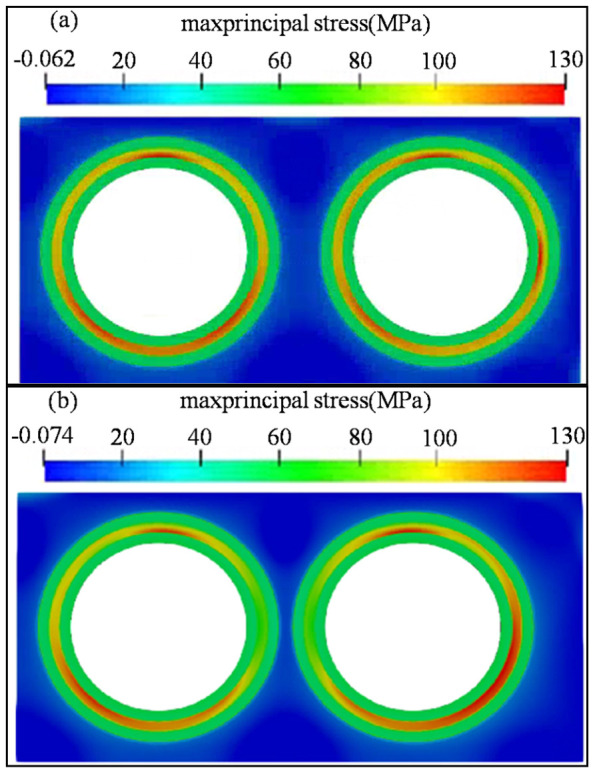
Maximum principal stress distribution in FCM fuel at a fast neutron fluence of 9.4 × 10^23^ n/m^2^: (**a**) Case 1, (**b**) Case 2.

Due to the complex structure and multi-interface characteristics of FCM fuel, the interface variable was calculated first to analyze the cracking behavior. By characterizing the interface locations between IPyC-SiC, SiC-OPyC, and OPyC-SiC matrix, the distributions of fracture strength and critical energy release rate for the FCM fuel were determined. The distribution of the maximum principal stress in FCM fuel under different particle spacings (Case 1 and Case 2) was investigated. In the early stage of irradiation (fast neutron fluence of 9.4 × 10^23^ n/m^2^), the maximum principal stress distribution of the FCM fuel is shown in Figure 2.

The SiC matrix was subjected to compressive stress, while the SiC layer was subjected to tensile stress. The influence of particle spacing on the principal stress was minimal. The maximum principal stress of the fuel particles appeared in the SiC layer, with a value of approximately 130 MPa, which was lower than the fracture strength of the SiC layer. Particle spacing affected the principal stress state of the SiC matrix between particles; a smaller particle spacing resulted in increased stress in the SiC matrix. This was primarily attributed to the matrix shear caused by particle deformation as the spacing decreased. Overall, at low burnup, the maximum principal stress of the FCM fuel was lower than the material fracture strength, and particle spacing had a minor influence on the maximum stress distribution state.

**Figure 3:**
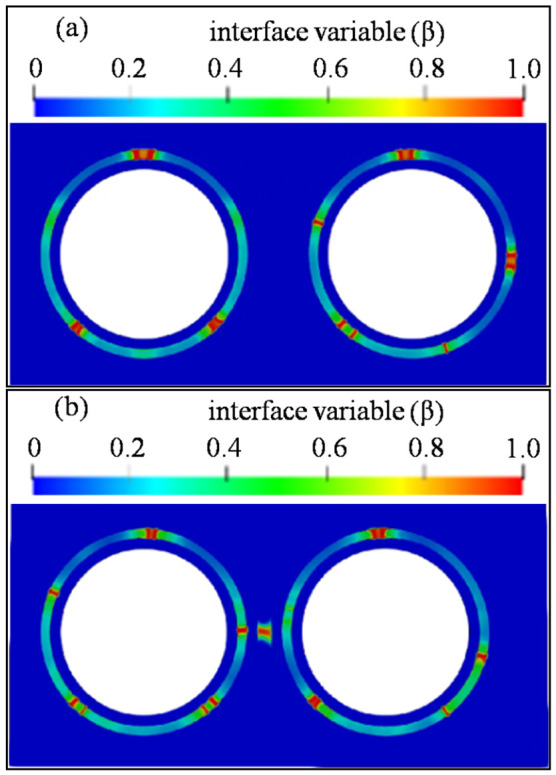
Cracking morphology of FCM fuel under a fast neutron fluence of 2.3 × 10^25^ n/m^2^: (**a**) Case 1, (**b**) Case 2.

With the increase in burnup, the internal pressure of fission gas in the FCM fuel gradually increased, leading to elevated hoop stresses in the coating layers. Concurrently, the irradiation deformation of the SiC matrix and the coating layers increased. As burnup increased, the significant deformation of the TRISO particles within the FCM fuel caused the particles to pull on the surrounding SiC matrix, resulting in increased hoop stress in the internal SiC matrix. This result, previously demonstrated in earlier calculations, shows that the maximum principal stresses in both the SiC layer and the SiC matrix increase sharply as the spacing between TRISO particles decreases [18]. In the case of smaller particle spacing, cracking occurred in the SiC matrix when the stress between the particles exceeded the fracture strength, whereas the SiC matrix maintained structural integrity in the case of larger spacing. When the fast neutron fluence reaches 3.3 × 10^24^ n/m^2^, the maximum principal stress in the SiC layer can reach 400 MPa. In contrast, for a larger particle spacing (Case 1), the maximum principal stress in the SiC layer remains below 300 MPa, which is below the fracture threshold. Figure 3 illustrates the cracking morphology of the FCM fuel at a fast neutron fluence of 2.3 × 10^25^ n/m^2^. A comparison indicates that a greater number of cracks were present in the SiC layer when the particle spacing was smaller. Under the condition of smaller TRISO particle spacing, cracking occurred in the SiC matrix, located between two particles, while the structures of the IPyC and OPyC layers remained intact.

**Figure 4:**
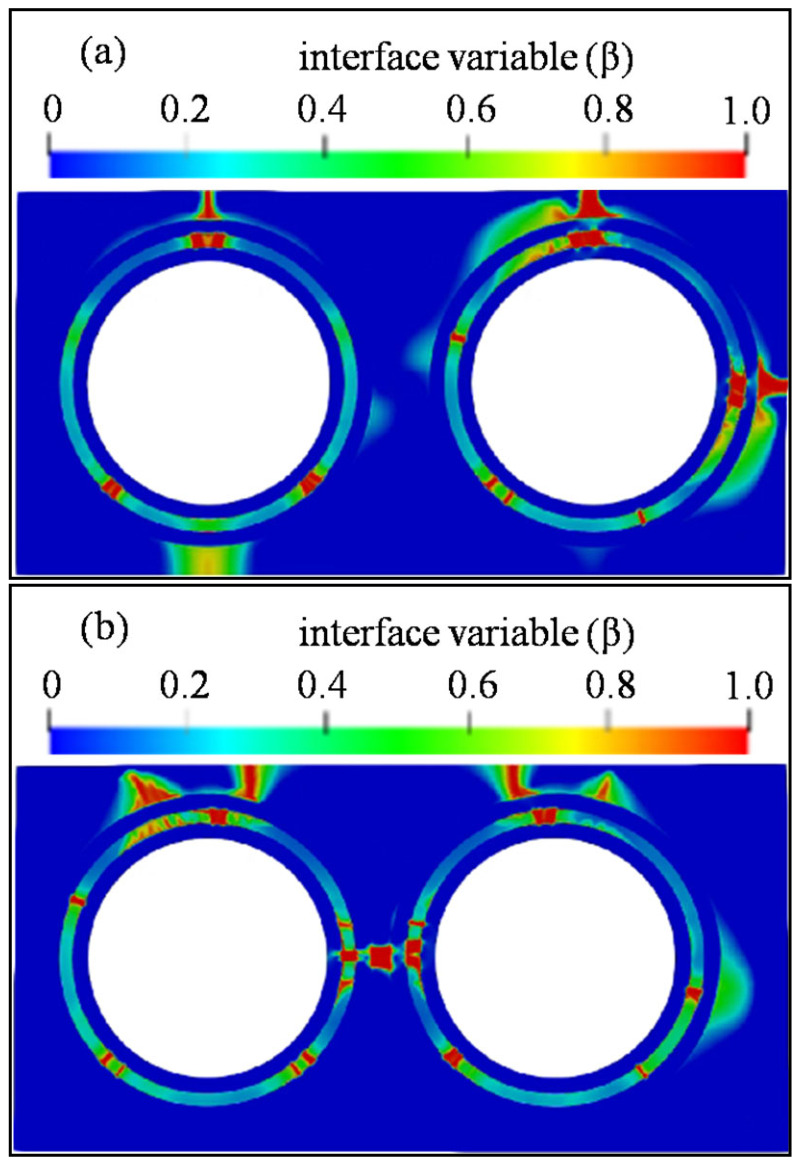
Cracking morphology of FCM fuel under a fast neutron fluence of 4.1 × 10^25^ n/m^2^: (**a**) Case 1, (**b**) Case 2.

For FCM fuel, the SiC matrix and PyC layers possessed a certain capacity to retain radioactive fission products; therefore, the structural integrity of the SiC matrix and PyC layers was essential to ensure the safety performance of the FCM fuel. According to Equations (17)–(19), the irradiation deformation and irradiation creep of the PyC layers continuously increased with the increase in fast neutron fluence. When the fast neutron fluence reached 4.1 × 10^25^ n/m^2^, the cracking morphology of the FCM fuel is shown in Figure 4.

At high fluence, cracking occurred in both the SiC matrix and the coating layers. This was primarily attributed to the continuous increase in the irradiation deformation of the SiC matrix and coating layers under high fluence, combined with the increased internal pressure of the fuel particles, which resulted in elevated stress in the matrix and coating layers. As observed in the figure, when the particle spacing was reduced, cracking occurred in the SiC matrix between the particles, and the cracks propagated through the inter-particle region. In contrast, for the FCM fuel with larger particle spacing, the SiC matrix between the particles remained intact, although cracking occurred in the SiC matrix at the fuel edge. Similarly, at the same burnup level, the FCM fuel with smaller particle spacing exhibited a greater number of cracks and a larger crack area fraction. Furthermore, the interconnection of matrix cracks between particles led to the loss of fission product retention capability.

### 4.2. Effect of Matrix Defect

**Figure 5:**
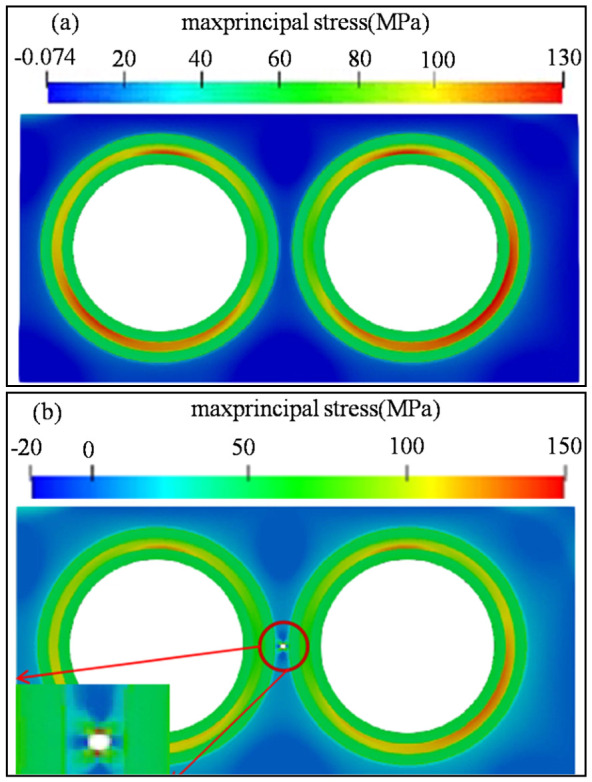
Maximum principal stress distribution in FCM fuel at a fast neutron fluence of 9.4 × 10^23^ n/m^2^: (**a**) Case 2, (**b**) Case 3.

FCM fuel is typically fabricated via high-temperature sintering, during which residual pore may persist in the SiC matrix due to its low sintering activity. This study compared Case 2 and Case 3 (particle spacing: 0.9 mm) to evaluate the influence of residual pore on the cracking behavior of FCM fuel. Figure 5 presents the distribution of maximum principal stress in FCM fuel with and without residual pore. At a fast neutron fluence of 9.4 × 10^23^ n/m^2^, the maximum principal stress in fuel without residual pore occurred in the SiC layer, whereas in fuel with residual pore, it localized in the matrix—reaching 150 MPa due to stress concentration at the pores, which exceeded the maximum stress in the SiC layer. Overall, the presence of residual pore in the SiC matrix induced localized stress concentration and significantly altered the distribution of maximum principal stress in FCM fuel.

**Figure 6:**
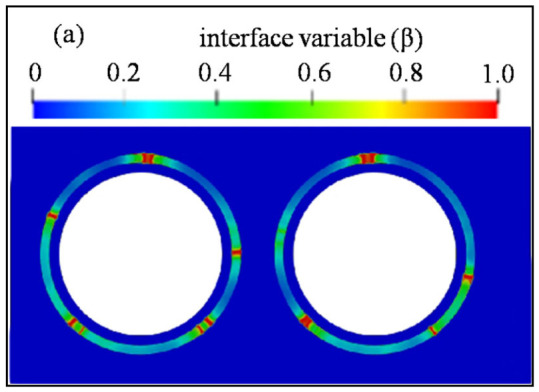

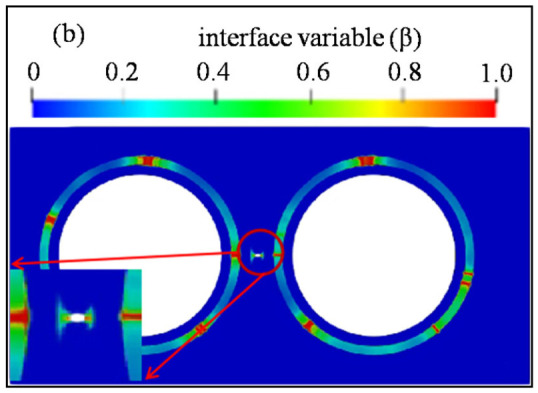
Cracking morphology of FCM fuel under a fast neutron fluence of 1.7 × 10^25^ n/m^2^: (**a**) Case 2, (**b**) Case 3.

Figure 6 illustrates the cracking state of FCM fuel with and without residual pore at a fast neutron fluence of 1.7 × 10^25^ n/m^2^. At this fluence, cracks formed in the SiC layer of fuel without residual pore, but the SiC matrix remained intact, as the matrix stress was insufficient to reach its fracture strength under low neutron fluence. Additionally, cracks in the coating layer deflected at the interface and did not propagate into the matrix. In contrast, in fuel with residual pore, stress concentration at the pores initiated cracking within the matrix, and corresponding cracks also appeared in the SiC layer. Under the same neutron fluence, FCM fuel with residual pore exhibited more extensive cracking in the SiC matrix and a higher number of cracks.

**Figure 7:**
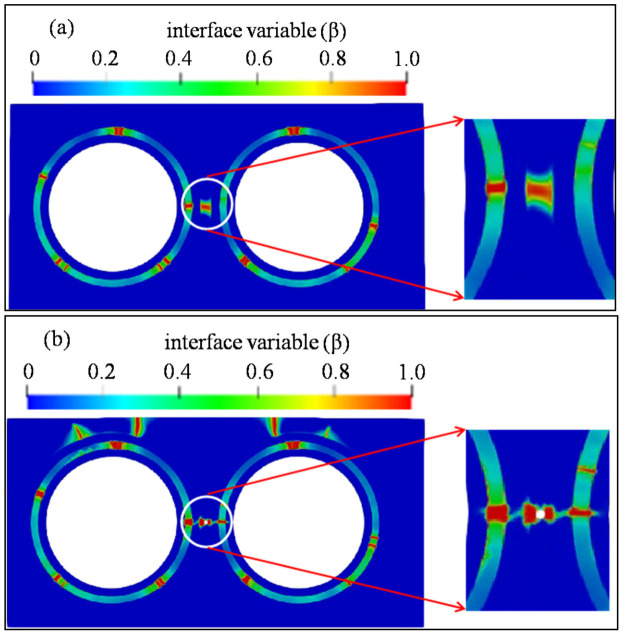
Cracking morphology of FCM fuel under a fast neutron fluence of 2.3 × 10^25^ n/m^2^: (**a**) Case 2, (**b**) Case 3.

With increasing burnup, at a fast neutron fluence of 2.3 × 10^25^ n/m^2^ (showed in Figure 7), cracks emerged in the matrix of fuel without residual pore, localized between two TRISO particles, while other regions of the matrix remained uncracked. This was attributed to deformation of TRISO particles during irradiation, inducing shear stress in the SiC matrix between particles and triggering crack initiation. In fuel containing residual pore, cracks appeared throughout the matrix at various locations, as the porosity altered the overall stress distribution. Moreover, cracks propagated through both the coating and matrix at pore sites, forming through-cracks. Such through-cracks in FCM fuel compromise the containment of radioactive fission products and degrade its safety performance.

## 5. Conclusions

This study employed the MOOSE V1.3 multiphysics coupling platform and the cohesive phase-field fracture theory to simulate cracking and crack propagation behavior in fully ceramic microencapsulated (FCM) fuel under representative operating conditions. Interface locations were explicitly represented by interface variables, and the influence of interfaces on the critical energy release rate and fracture strength was incorporated to properly model material interfaces. Crack initiation and propagation were analyzed for a representative FCM fuel structure containing two TRISO particles. Results showed that, when the OPyC layer was tightly bonded to the SiC matrix, the SiC layer experienced high tensile stress while the matrix was subjected to compressive stress. In the presence of residual pore within the SiC matrix, significant stress concentration occurred at the porosity sites, substantially altering the overall stress state of the FCM fuel. As the fast neutron fluence increased, closely spaced TRISO particles promoted cracking in the matrix between them, and the number of cracks in the SiC layer increased markedly under the same fluence. With further increase in neutron fluence, microvoids and other defects within the matrix served as crack nucleation sites; cracks gradually propagated toward the OPyC layer, ultimately leading to through-thickness cracking in both the coating and the matrix.

## Figures and Tables

**Table 1:** Case settings for sensitivity analysis of FCM fuel.

Case ID	Case 1	Case 2	Case 3
TRISO particle Spacing	1 mm	0.9 mm	0.9 mm
Defect	None	None	0.3 mm Residual pore

**Table 2:** The critical energy release rate and fracture strength of the materials.

Materials Properties	PyC	SiC	IPyC-SiC	SiC-OPyC	OPyC-Matrix
critical energy release rate (N/m)	400	20	20	20	20
fracture strength (MPa)	218	350	200	200	77

## Data Availability

The original contributions presented in this study are included in the article. Further inquiries can be directed to the corresponding authors.
